# Molecular characterization of megaplasmids encoding the type VI secretion system in *Campylobacter jejuni* isolated from chicken livers and gizzards

**DOI:** 10.1038/s41598-020-69155-z

**Published:** 2020-07-27

**Authors:** Daya Marasini, Anand B. Karki, John M. Bryant, Robert J. Sheaff, Mohamed K. Fakhr

**Affiliations:** 1 Department of Biological Science, The University of Tulsa, Tulsa, OK USA; 2 Department of Chemistry and Biochemistry, The University of Tulsa, Tulsa, OK USA

**Keywords:** Food microbiology, Microbiology, Environmental microbiology

## Abstract

Megaplasmids in *Campylobacter* spp. likely play important roles in antibiotic resistance, virulence, and horizontal gene transfer. In this study, megaplasmids pCJDM202 (119 kb) and pCJDM67L (116 kb) from *C. jejuni* strains WP2-202 and OD2-67, respectively, were sequenced and characterized. These megaplasmids contained genes for tetracycline resistance [*tet*(O)], the Type IV secretion system, conjugative transfer and the Type VI secretion system (T6SS). The T6SS genes in *Campylobacter* plasmids encoded genes and proteins that were similar to those identified in *Campylobacter* chromosomal DNA. When the megaplasmid pCJDM202 from *C. jejuni* WP2-202 was transferred via conjugation to *C. jejuni* NCTC11168 Nal^+^, transconconjugants acquired tetracycline resistance and enhanced cytotoxicity towards red blood cells. A T6SS mutant of strain WP2-202 was generated and designated *Δhcp3*; the mutant was significantly impaired in its ability to lyse red blood cells and survive in defibrinated blood. The cytotoxicity of *Campylobacter* strains towards the human embryonic kidney cell line HEK 293 was not impacted by the T6SS. In summary, the T6SS encoded by *Campylobacter* megaplasmids mediates lysis of RBCs and likely contributes to survival on retail meats where blood cells are abundant.

## Introduction

The high prevalence of *Campylobacter* spp. in retail chicken products is associated with campylobacteriosis outbreaks worldwide^1^ and reflects the challenges in preventing *Campylobacter* contamination in retail meat production and storage^2,3^. The consumption of undercooked liver dishes prepared from contaminated chicken liver products was responsible for the multistate outbreaks of campylobacteriosis in the USA^4–6^. The antimicrobial resistance and virulence genes in *Campylobacter* have substantially increased the difficulty in controlling campylobacteriosis^7^, and the transmission of drug-resistant foodborne pathogens through livestock, food products and humans creates a huge public health burden^8^. Various aspects of *Campylobacter* contamination, transmission, and pathogenicity remain unexplored^9^.

The genetic transfer of antimicrobial resistance and virulence genes between organisms in environmental niches is a common route of acquisition by foodborne pathogens^8^. Plasmids in foodborne pathogens such as *Campylobacter* often encode antimicrobial resistance genes such as tetracycline resistance (*tetO*); furthermore, pVir plasmids in *Campylobacter* are frequently involved in virulence^10,11^. Many *Campylobacter* plasmid sequences have been deposited in GenBank^12,13^, and these plasmids have been categorized according to size and genomic composition^14^. The complete sequences for several *C. jejuni* megaplasmids are available but the potential role of these genes in virulence and survival is not clear^13^.

The Type VI secretion system (T6SS) has been identified in diverse species of Gram-negative bacteria and functions to kill competing bacteria via a bacteriophage-like invasion and injection mechanism^15,16^. Various foodborne pathogens, including *Salmonella* spp*., Escherichia coli* and *Campylobacter* spp*.,* possess the T6SS^17,18^. The T6SS in *Campylobacter* was similar to the protein products encoded by the pathogenicity island in *Helicobacter hepaticus*^19^. Previous studies demonstrated that the *Campylobacter* T6SS was responsible for erythrocyte cytotoxicity, host cell adherence and colonization^19,20^. Among 13 T6SS genes in various isolates of *C. jejuni* and *C. coli*, *hcp* had a major role in virulence^21^; furthermore *hcp* overexpression enhanced the invasiveness and adherence of *C. jejuni*^20^.

Although the T6SS system has been identified in various foodborne and clinical pathogens, very few plasmids are known to harbor a complete set of T6SS genes^12,13,22,23^. Since plasmids play a major role in horizontal gene transfer, the potential transfer of T6SS genes among foodborne pathogens is possible; furthermore, horizontal transfer of T6SS genes was recently demonstrated in *Vibrio alginolyticus*^24^. However, it is important to mention that *Campylobacter* spp. do not readily exchange genetic material with other bacteria. The existence of restriction barriers and genes such as *Cj1051* in *C. jejuni* can prevent the incorporation of foreign bacteriophage particles into *Campylobacter* genomes^25^. In general, *Campylobacter* plasmids exhibit very limited homology to plasmid DNA in other gram-negative species^12,26^. However, the plasmid-encoded tetracycline resistance genes in *Campylobacter* and *Streptococcus* show similarity, which indicates possible genetic exchange between these gram-negative and gram-positive genera^27^. With the exception of previous studies on the pVir plasmid of *C. jejuni*, there is little information about the role of large plasmids in *C. jejuni* and *Campylobacter coli*^11^. In this study, we characterized two megaplasmids in *C. jejuni* isolates from chicken livers and gizzards; both megaplasmids encoded the type IV (T4SS) and type VI secretion systems. The relatedness of these plasmids with other *Campylobacter* plasmids encoding the T6SS was evaluated, and the potential role of the plasmid-encoded T6SS in transfer and survival was investigated.

## Materials and methods

### Bacterial strains and plasmids

Pulsed-field gel electrophoresis (PFGE) was previously utilized to show that *C. jejuni* strains WP2-202 and OD2-67 harbor megaplasmids^28^. Whole genome isolation and sequence analysis of these strains were briefly reported in a genome announcement^12^. Genome size, G + C content, number of genes and accession numbers are listed in Table 1. The megaplasmids pCJDM67L (accession no. CP014745) and pCJDM202 (accession no. CP014743) isolated from *C. jejuni* strains OD2-6*7* and WP2-202 were used for genomic comparison and characterization. Bacterial strains were grown in Mueller–Hinton blood (MHB) agar and MHB broth containing 16 µg/ml of tetracycline under micro-aerobic conditions for 72 h. *C. jejuni* NCTC11168 (Nal +), a nalidixic acid resistant (Nal^R^) variant from our laboratory, was used for conjugation and transformation experiments.

**Table 1:** Genomic features of *C. jejuni* strains OD2-67 and WP2-202 and their plasmids.

Strains	Plasmids	Chromosome size (bp)	Plasmid size (bp)	Genes	Pseudogenes	RNA	G + C	Accession number
*C. jejuni* WP2 202		1,681,907	–	1781	42	56	30.5	CP014742
	pCJDM202		119,543	116	11	–	27.2	CP014743
*C. jejuni* OD2 67		1,672,837	–	1786	64	54	30.5	CP014744
	pCJDM67L		116,833	108	9	–	26.9	CP014745
	pCJDM67S		36,603	46	1	–	26.1	CP014746

### Whole genome and plasmid sequencing

The isolation of genomic and plasmid DNA and sequence analysis was performed as described previously^12^. Briefly, *Campylobacter* cells were transferred to MHB broth and grown for 72 h with vigorous shaking (175 rpm) at 42 °C. Cells were harvested after centrifugation and genomic DNA was isolated using the DNeasy Blood and Tissue Kit (Qiagen Inc., Valencia, CA). Plasmids were isolated using the Qiagen plasmid midi-kit as recommended by the manufacturer. Library preparation for whole genome and plasmid sequencing was conducted using the Nextera XT sample preparation kit according to manufacturer’s instructions. Prepared libraries were sequenced in the Illumina Miseq platform with the Illumina MiSeq V2 reagent kit 2 × 150 cycles (Illumina Inc., CA). Sequence assembly was performed in the CLC Genomic Workbench v. 7.5.1 (Qiagen Inc.) Plasmid sequences were annotated using the RAST online tool (https://rast.nmpdr.org/rast.cgi)^29^ and the NCBI Prokaryotic Genome Annotation Pipeline.

### Comparative genomic analysis

Comparison of genomic DNAs from *C. jejuni* strains WP2-202 (Gene bank accession no. CP014742) , OD2-67 (CP014744) and NCTC11168 (AL111168.1) was performed with GView (https://server.gview.ca/). Sequences for the following *Campylobacter* plasmids with T6SSs were downloaded from GenBank on 11/14/2018 (accession numbers are shown in parenthesis): pCJDM202 (CP014743); pCJDM67L (CP014745); pMTVDSCj13-2 (CP017417); pCOS502 (CP018901); pCOS503 (CP025282); pCC14983A-1 (CP017026), and a plasmid from *C. jejuni* strain RM3194 (CP014345). Pangenome and core genome analysis were carried out with GView. The BLAST nucleotide analysis used in GView had e-values < 1e−10, alignment length cutoff values = 100 and percent identity cutoff values = 80 .

### Conjugation experiments

*C. jejuni* WP2-202 contains a single, T6SS-containing tetracycline-resistance (Tet^R^) megaplasmid (pCJDM202) and was selected as a donor in conjugation experiments. The recipient strain *C. jejuni* NCTC11168 Nal^+^ was isolated by inoculating a bacterial suspension (~ 10^8^ cells) to MHB agar containing 30 µg/ml of nalidixic acid. After 3 days, Nal^R^ colonies were further selected on MHB agar containing 60 µg/ml nalidixic acid. Heat-enhanced conjugation was performed as described previously^30^. Briefly, a 72-h culture of the recipient NCTC11168 Nal^+^ (~ 10^8^ cells) was incubated at 50 °C for 30 min and allowed to cool at 25 °C for 3 min; recipient cells (0.5 ml) were then mixed with 0.5 ml of the donor *C. jejuni* WP2-202 (~ 10^8^ cells; 72-h culture); 25 µl of the mixture was inoculated to MHB agar. After a 48-h incubation period, cells from inoculation sites were removed from the agar plates and suspended in 0.5 ml MH broth. Transconjugants were selected on MHB agar containing nalidixic acid and tetracycline at 60 and 16 µg/ml, respectively.

Plasmid transfer in putative transconjugants was confirmed by PFGE as described previously^28^. Briefly, agar plugs were digested with *Sma*I and S1 nuclease and subjected to PFGE for 16 h^28^. The presence of the T6SS in selected transconjugants was confirmed by PCR using primer pairs. Transconjugants TCF8 and TCF11 were used in further experiments.

### Construction of the *hcp* mutant

The *cat* gene, which encodes chloramphenicol acetyltransferase and confers chloramphenicol resistance (Cm^R^), was used to disrupt *hcp*. The *cat* cassette with its native promoter (798 bp) was inserted between nucleotides 60 and 460 in the *hcp* coding region, thus deleting a 400 bp fragment. The mutated *hcp* was constructed and cloned in pBluescript SK + (pBSK + *Δhcp*) as a commercial service provided by Biomatik USA (Wilmington, Delaware).

Electroporation was used to introduce the mutant form of *hcp* into *C. jejuni* WP2-202. To prepare cells for electroporation, WP2-202 was initially cultured on MHB agar at 42 °C for 48 h in microaerobic conditions; cells were then removed and suspended in 1 ml MHB broth. After centrifugation at 10,000 rpm for 5 min, the supernatant was discarded and the pellet was resuspended in 100 µl MHB; the suspension was then inoculated to MHB agar as a lawn and incubated for 24 h at 42 °C in microaerobic conditions. Cells harvested from the lawn were suspended in 1 ml MHB, pelleted by centrifugation, and washed four times with 272 mM sucrose containing 15% glycerol. Cells were then resuspended in 272 mM sucrose containing 15% glycerol and 50 µl aliquots were stored at − 70 °C.

Frozen WP2-202 cells were thawed on ice, and 2 µl of pBluescript SK + containing the mutant *hcp* gene (pBSK + *Δhcp*) was added. This mixture was then subjected to electroporation in a Gene Pulser III (Bio-Rad, USA) at 2.5 kV, 25 µF and 200 Ohms. Electroporation was also used to introduce a linear DNA fragment containing the synthetic mutated *hcp*; this was amplified using pBSK + *Δhcp*) by the primers P1 and P4 (Table S1), and a 2 µl amplified PCR product was introduced into electrocompetent cells. After electroporation, WP2-202 cells were quickly resuspended in 100 µl of SOC broth, inoculated to MHB agar, and incubated at microaerobic conditions at 37 °C for 24 h. Transformants were selected on MHB agar containing chloramphenicol (20 µg/ml) and tetracycline (16 µg/ml). Putative mutants containing deletions in *hcp* were screened using primers P5 (upstream of *hcp*) and P6 (downstream of *hcp*) and various combinations of primers P1, P2, P3, and P4 (Table S1); potential mutants were further confirmed by sequencing. A *hcp* mutant designated *Δhcp3* was selected for further experiments.

### Complementation experiments

For complementation, cloning vector pRY108^31^ and PCR-amplified *hcp* by primers Hcp-com-F and Hcp-com-R (Table S1) from pCJDM202 were digested with *Bam*HI/*Xba*I and ligated. The resulting constructs were used to transform *E. coli* DH5α and selected on Luria–Bertani (LB) agar containing 100 µg/ml kanamycin; gel electrophoresis was used to confirm *E. coli* transformants containing *hcp*. Prior to transformation, *C. jejuni Δhcp3* cells were grown on MHB agar containing chloramphenicol (20 µg/ml) and tetracycline (16 µg/ml) for 48 h at 42 °C in microaerobic conditions. Multiple attempts were undertaken to introduce *hcp* into the *C. jejuni Δhcp3* mutant by electroporation, and transformants were screened on MHB agar containing chloramphenicol (20 µg/ml), tetracycline (16 µg/ml), and kanamycin (100 µg/ml).

### Assays for confirmation of functional T6SS in megaplasmid pCJDM202

Cytotoxicity assay, survival in blood and human cell viability assay of wild type strain carrying megaplasmid pCJDM202 (*C. jejuni* WP2-202), *hcp* gene mutant (*C. jejuni Δhcp3*) and transconjugants were carried out as described previously^19^ with few modifications. These assays were used in our study for the confirmation of a functional T6SS found in megaplasmid pCJDM202 as similar experiment was used in a previous study^19^ to characterize functional T6SS in the chromosome of *C. jejuni* 108.

### a. Cytotoxicity assays

*C. jejuni* NCTC11168 Nal^+^, transconjugants TCF8 and TCF11, WP2-202, and *Δhcp3* (*hcp* mutant) were grown on MHB agar for 72 h and transferred to liquid media for an additional 48 h-incubation at 42 °C in microaerobic conditions. The wild-type WP2-202 was grown in MHB agar with tetracycline (32 µg/ml), whereas *Δhcp3* was grown in MHB agar containing tetracycline (32 µg/ml) and chloramphenicol (20 µg/ml) for 72 h and 7 days, respectively. The extracted cells were then pelleted and resuspended in fresh MHB broth, and the OD_600_ was adjusted to 1.5. Red blood cells (RBCs) were pelleted from 12.5 µl defibrinated horse blood (Hemostat, Dixon, CA) by centrifugation at 10,000 rpm for 5 min, and the supernatant was discarded. The pelleted RBCs were resuspended in 250 µl PBS containing 0.4 mM CaCl_2_^19^. Bacterial suspensions (1 ml) were added to the blood cells and incubated microaerobically at 42 °C for 6 h. After six hours, the A_420_ was measured to quantify the degree of hemolysis. Water and PBS were used as positive and negative controls, respectively. All experiments were carried out at least twice with technical triplicates.

### b. Survival of *C. jejuni* in blood

WP2-202 and *Δhcp3* cells from 18-h cultures grown at 42 °C on MHB agar in microaerobic conditions were harvested and pelleted. Cell suspensions (A_600_ ~ 0.1) were prepared in PBS (pH 7.34), and 100 μl aliquots were inoculated into 10 ml defibrinated horse blood (Hemostat, CA) and incubated in 50 mm petri dishes (microaerobic conditions, 72 h, 42 °C). Samples (100 μl) were removed at 0, 24, 48, and 72 h, and viable cells counts were determined. Experiments were performed in triplicate. One end t-test was carried out for statistical analysis of all the experimental data.

### c. Human cell viability assay

The human embryonic kidney (HEK 293) cell line [American Type Culture Collection (ATCC), Manassas, VA, USA] was used to study the effect of plasmid pCJDM202 on cell cytotoxicity. Three-day-old liquid cultures of *C. jejuni* NCTC11168 Nal^+^, TCF8, TCF11, WP2-202 and *Δhcp3* were obtained from cells grown in MHB (microaerobic conditions, 42 °C) and separated by centrifugation into bacterial cell and supernatant fractions. Various concentrations of each strain were incubated with HEK 293 cells at 37 °C in 5% CO_2_ for 6 h, and cell viability was determined^32^. The Cell-Titer Blue assay (Promega, Madison, WI) was used to measure viability; in this assay, living cells convert resazurin to the fluorescent resorufin, and the signal is measured with a TECAN Safire plate reader.

### Tetracycline resistance

*C. jejuni* WP2-202 and transconjugants TCF8 and TCF11 and were grown micro-aerobically on MHA plates containing tetracycline at 1, 2, 4, 8, 16, 32, 64, 128, 256 and 512 µg/ml, and the growth of strains was measured after 48 h.

## Results

### Comparative genomics of *C. jejuni* WP2-202 and OD2-67

**Figure 1:**
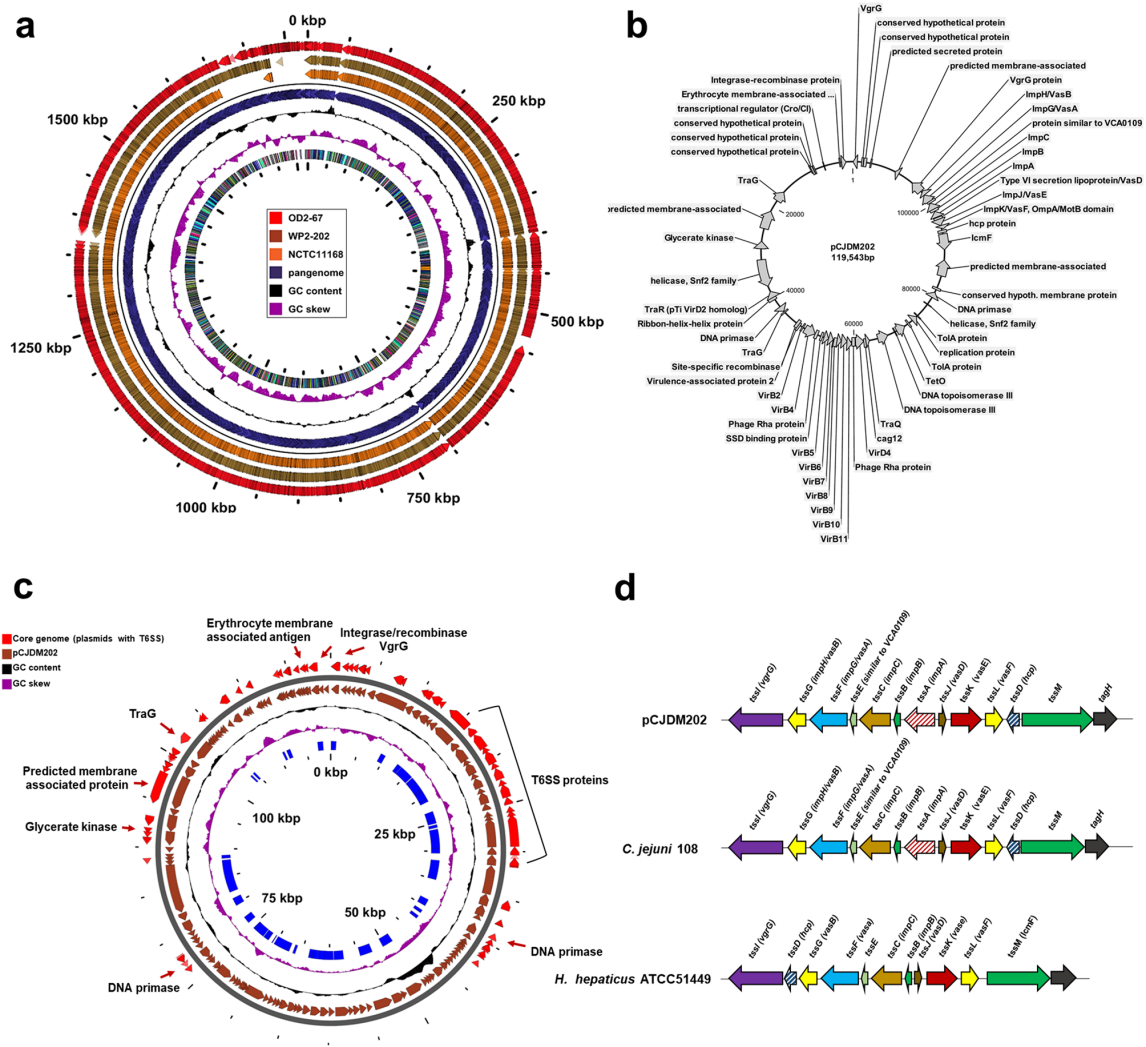
(**a**) Pangenome analysis of the chromosomal DNA in *C. jejuni* strains NCTC11168, WP2-202 and OD2-67. (**b**) Functional map of the genes encoded by the megaplasmid pCJDM202. (**c**) Core genome analysis of all *Campylobacter* plasmids encoding the T6SS. (**d**) Organization of T6SS genes encoded by pCJDM202, *C. jejuni* strain 108 and *H. hepaticus* ATCC51449. The organization of the T6SS gene cluster encoded by pCJDM202 was conserved in all *C. jejuni* and *C. coli* plasmids.

The genome sizes of *C. jejuni* WP2-202 and OD2-67 were similar to other sequenced *C. jejuni* strains, which ranged from 1.6–1.8 Mb in size^13,22^. Overall, the sequences of these two genomes shared a great deal of homology, and the organization of ORFs was similar. Chromosomally-encoded genes in the two strains were highly homolgous (99–100% sequence identity). *C. jejuni* WP2-202 and OD2-67 lacked a few transferase genes that were present in the reference strain NCTC11168; other differences between these two strains (WP2-202 and OD2-67) included hypothetical and phage-related proteins (Fig. 1a). Detailed results of BLAST analysis and differences in coding sequences (CDS) are shown in Table S2.

### Plasmids harboring the T6SS

*C. jejuni* WP2-202 contains a 119,543-bp megaplasmid designated pCJDM202, whereas OD2-67 contains pCJDM67L and pCJDM67S, which are 116,833 and 36,603 bp, respectively (Table 1). The smaller plasmid, pCJDM67S, shares homology with the previously identified virulence plasmid, pVir^11^. Blast analysis revealed that the two megaplasmids, pCJDM202 and pCJDM67L, have similar genomic compositions and a high level of sequence homology (Table S3). A few hypothetical proteins were identified that distinguished the two plasmids, and detailed differences are shown in Table S3. pCJDM202 and pCJDM67L contain genes encoding the T6SS, the type IV secretion system (T4SS), conjugative transfer and tetracycline resistance (Fig. 1b).

**Figure 2:**
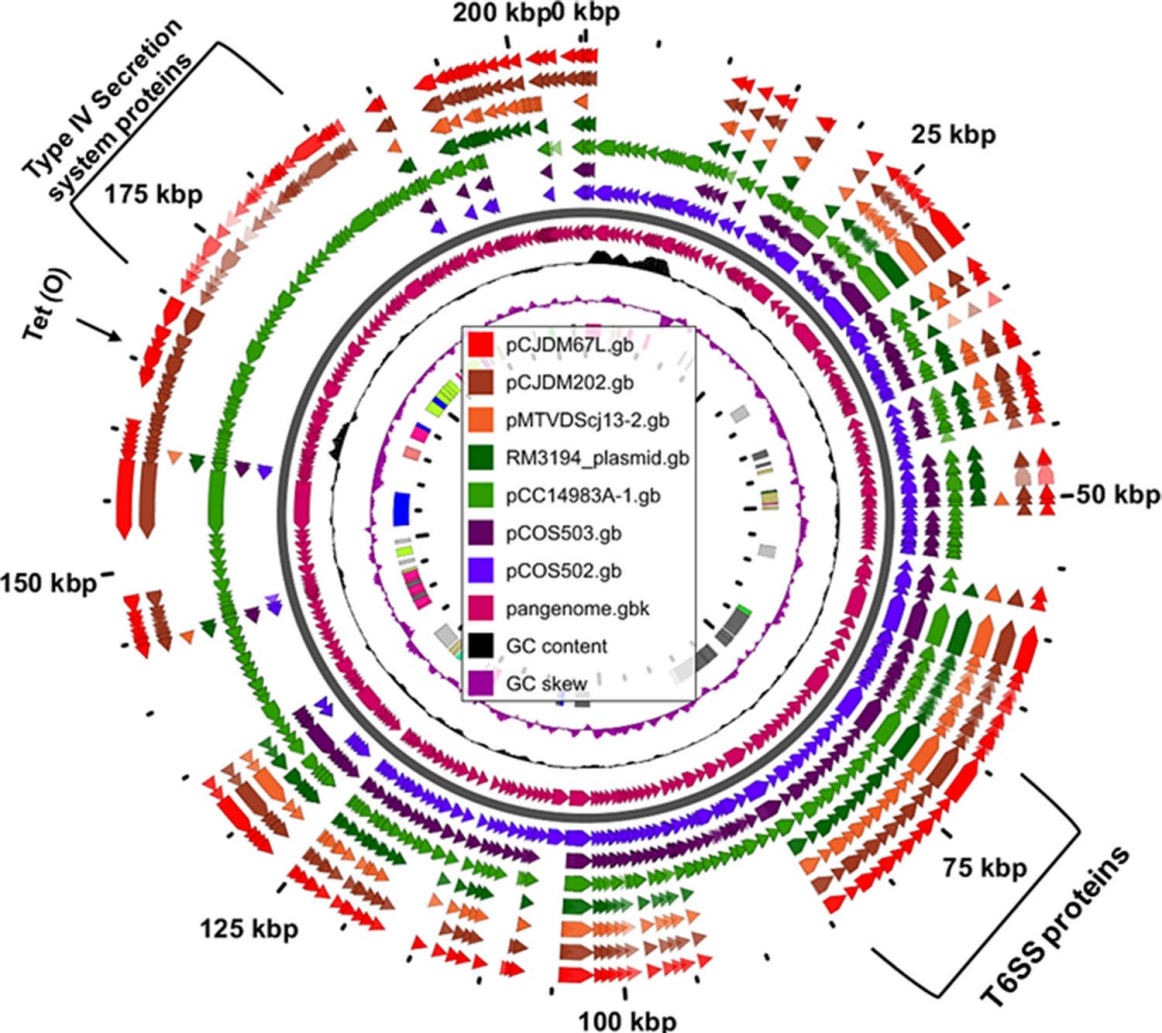
Pangenome analysis of *Campylobacter* plasmids harboring the T6SS.

Analysis of the *C. jejuni* and *C. coli* plasmids harboring the T6SS in Genbank showed the presence of 13 conserved T6SS genes in all plasmids (Table S4; Fig. 1c, 1d). The genomic arrangement of T6SS genes found in megaplasmids (for example: pCJDM202) was similar to the T6SS cluster in the genome of *C. jejuni* 108, however it differed slightly from the T6SS in *Helicobacter hepaticus* ATCC 51,449^19^ (Fig. 1d). pCJDM67L and pCJDM202 encoded T4SS genes and *tet(O)*, which are also borne on the *C. coli* plasmid pCC14983A-1(Fig. 2) (Table S5). Genes encoding the T4SS, conjugative transfer and tetracycline resistance were not common to all plasmids.

### Transfer of *C. jejuni* megaplasmids via conjugation

Heat shock was used to induce conjugal transfer of megaplasmid pCJDM202 from the donor WP2-202 to the recipient NCTC11168 Nal^+^. The presence of pCJDM202 in transconjugants TCF8 and TCF11 was confirmed by PFGE. The *Sma*I digest shows an extra fragment in the donor and transconjugants that was not present in NCTC11168 Nal^+^. The presence of the T6SS gene, *hcp*, was confirmed by PCR amplification of amplicon size of 1,420-bp in the transconjugants with primers P1 and P4 (Table S1).

### Mutagenesis and complementation

Homologous recombination was used to delete a 400-bp region of *hcp* and replace it with *cat*, which encodes Cm^R^. The mutant *Δhcp3* was confirmed by PCR and sequence analysis using primers P5 and P6 and various combinations of primers P1-P4, C1 and C2 (Table S1). PFGE was used to confirm that pCJDM202 was present in the *Δhcp3* mutant. Complementation of the *C. jejuni Δhcp3* mutant involved attempts to introduce pRY108 containing *hcp* into the mutant by electroporation. All attempts to transform the mutant were unsuccessful.

### Cytotoxicity, survival in blood cells, and human cell viability assay

**Figure 3:**
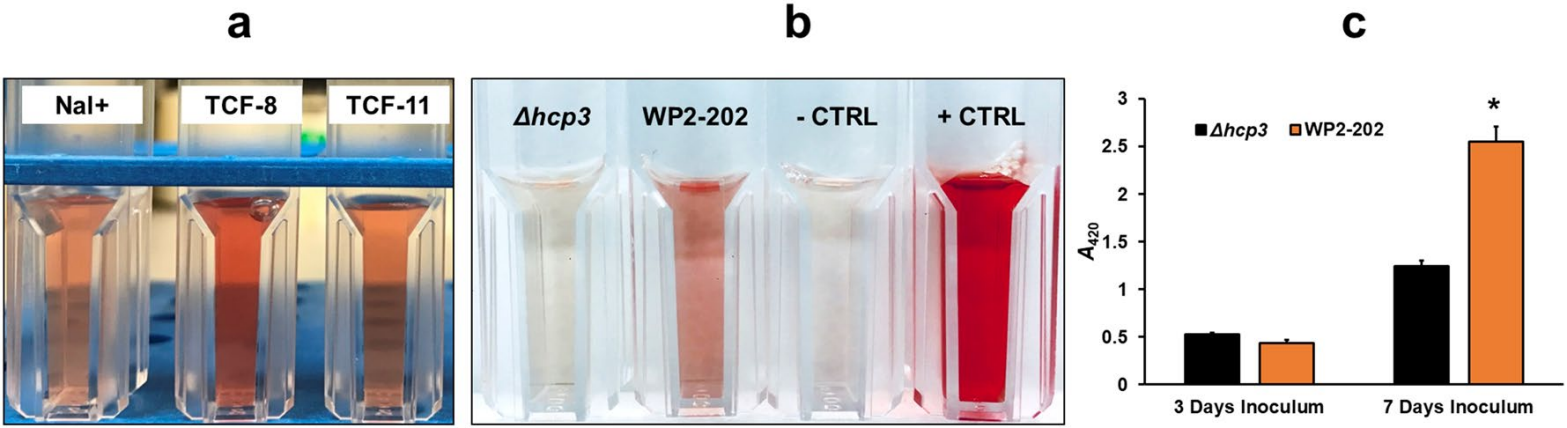
Hemolysis of red blood cells by *C. jejuni* strains. Suspensions with darker coloration have a high level of hemolytic activity. (**a**) Hemolytic activity in seven-day-old cultures of *C. jejuni* NCTC11168 Nal^+^ and transconjugants TCF8 and TCF11 containing pCJDM202. (**b**) Hemolytic activity in seven-day-old cultures of *C. jejuni* WP2-202 and the *Δhcp3* mutant. PBS and water were used as negative and positive controls. (**c**) Diagram showing the absorbance (*A*_420_) of supernatants after incubating red blood cells with bacterial suspensions of *C. jejuni* WP2-202 and the *Δhcp3* mutant. Column labeled with an asterisk (*) is significant at *P* < 0.05.

**Figure 4:**
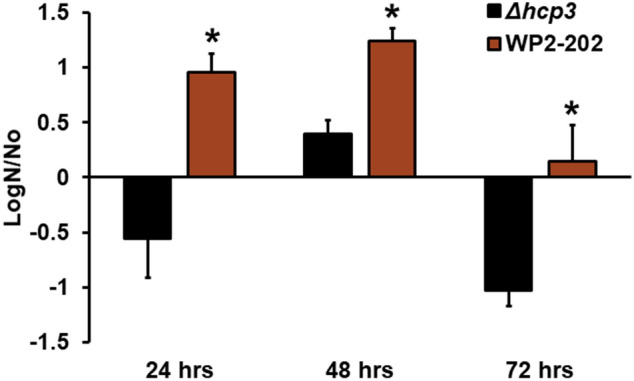
Survival of the *C. jejuni* WP2-202 and the *Δhcp3* mutant in defibrinated fresh horse blood. Error bars represent standard error of the mean (LogN/No). Columns labeled with an asterisk (*) represent significance at *P* < 0.05.

Transconjugants TCF8 and TCF11 were slightly more hemolytic than the recipient NCTC11168 Nal^+^, which lacks pCJDM202 (Fig. 3a). *C. jejuni* WP2-202 and the *Δhcp3* mutant showed similar hemolytic activity at three days (Fig. 3c), but *Δhcp3* showed significantly less hemolysis than the wild-type WP2-202 at seven days (Fig. 3b, c). When compared to the wild-type WP2-202, the *Δhcp3* mutant showed a significant decrease in survival when incubated in defibrinated horse blood (Fig. 4). Survival of the wild-type WP2-202 was significantly higher (*P* < 0.05) than *Δhcp3* at 24, 48, and 72 h (Fig. 4).

**Figure 5:**
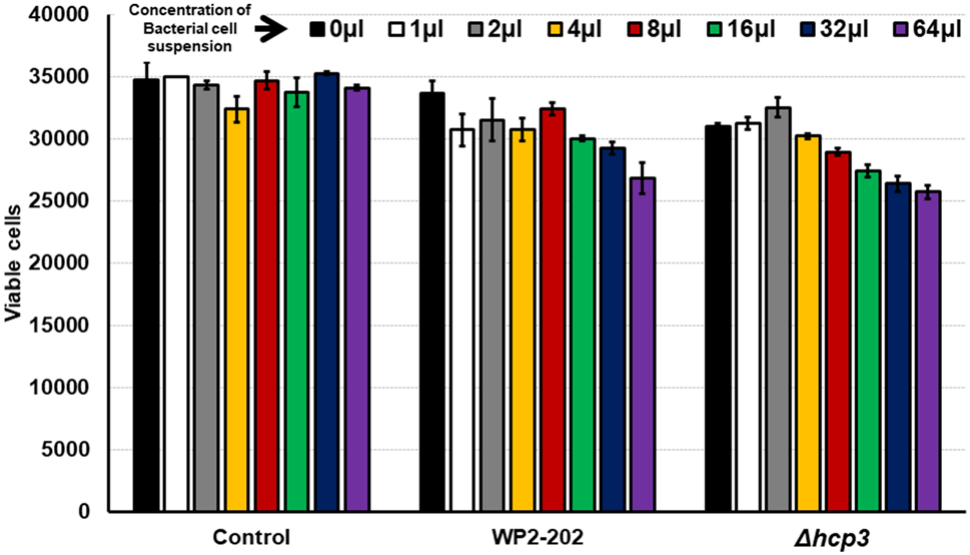
Viability of HEK 293 cells after a 6-h incubation with *C. jejuni* WP2-202 and the *Δhcp3* mutant. Viability was measured using the resazurin assay as described in Methods.

*C. jejuni* NCTC11168 Nal^+^ and transconjugants TCF8 and TCF11 did not differ significantly in their ability to cause lysis of HEK 293 cells (Fig. S1). Similarly, both *C. jejuni* WP2-202 and the *Δhcp3* mutant showed no significant differences in cytotoxicity relative to the control when incubated with HEK 293 cells (Fig. 5).

### Tetracycline resistance

*C. jejuni* NCTC11168 Nal^+^ was highly susceptible to tetracycline, and growth was inhibited beginning at 2 µg/ml (data not shown). Transconjugants TCF8 and TCF11 containing pCJDM202 showed a much greater tolerance to tetracycline (up to 256 µg/ml); this level is much higher than the donor WP2-202, which showed a MIC = 64 µg/ml*.*

## Discussion

The sequenced genomes of *C. jejuni* WP2-202 (isolated from chicken liver) and *C. jejuni* OD2-67 (chicken gizzard) revealed the presence of circular chromosomes and megaplasmids pCJDM202 and pCJDM67L respectively. The genome sizes of WP2-202 and OD2-67 were similar to strains isolated from retail meats and clinical sources^13,22,33^ and were highly homologous to reference strain NCTC11168. All three genomes harbored the multidrug efflux pump encoded by *cmeABC*, which has been characterized in other *C. jejuni* strains^34,35^. Both WP2-202 and OD2-67 encoded virulence factors that were present in NCTC11168, which indicates the potential pathogenicity of these strains. It is important to mention that campylobacteriosis outbreaks have been associated with poultry liver products, and the incubation time of campylobacteriosis originating from these products was short^36^.

*Campylobacter* spp. harbor plasmids that vary in size^14^; however, few *Campylobacter* strains possess megaplasmids^13,22,23^. The megaplasmids characterized in this study, pCJDM202 and pCJDM67L, are similar to the pTet plasmids and encode conserved proteins for conjugation and the T4SS^10^. Plasmids pCJDM67L and pCJDM202 encoded tetracycline resistance; other antibiotic resistance genes were not identified in the megaplasmids. However, megaplasmids from *C. jejuni* (turkey isolates) contained genes encoding aminoglycoside resistance^13^. Megaplasmids pCJDM67L and pCJDM202 also encode the T6SS, and core genome analysis of selected *C. jejuni/coli* plasmids revealed the presence of 13 conserved T6SS genes. These megaplasmids also harbor genes encoding DNA primase, erythrocyte-associated membrane protein, TraG, glycerate kinase and hypothetical proteins. Based on the conserved structure among these plasmids, we predict that additional, novel sequences were acquired from other sources (e.g. prophages, pathogenicity islands, secretion systems) and/or retained by selected plasmids. Pangenome analysis revealed various CDS that differed among *C. jejuni* and *C. coli* plasmids and showed species-specific clustering of plasmids with the T6SS. Plasmids pCJDM202, pCJDM67L and pCC14983A-1 (*C. coli* plasmid) are pTet-like plasmids that harbor *tet(O)* and the T4SS. We previously demonstrated that Type 1 (pTet), Type 2 and Type 3 plasmids in *Campylobacter* spp. were primarily of *C. jejuni/coli* origin^14^. However, further analyses with additional megaplasmid sequences is needed to conclusively show species-specific clustering since only seven megaplasmid sequences were used in the current study.

Our results show that megaplasmid pCJDM202 was conjugally transferred from *C. jejuni* WP2-202 to NCTC11168 Nal^+^, which indicates that the T6SS borne on pCJDM202 was mobilized between the two strains. In addition to an extrachromosomal location, various reports have documented the presence of the T6SS in the chromosomes of *C. jejuni* and *C. coli*^18,21^. The organization and relatedness of T6SS genes is conserved in *Campylobacter* plasmid (this study) and chromosomal DNA^19,20,37^, which supports the transmissibilty of the T6SS in *Campylobacter* spp. The transfer of pTet plasmids and *tet(O)* in *Campylobacter* spp. is well-documented^26,38^, and the acquisition of tetracycline and kanamycin resistance had been reported via natural transformation of *C. jejuni* in mixed populations of *Campylobacter*^39^; the latter might be enhanced in the biofilms that form during retail meat processing and storage^40^. Plasmids pCJDM202, pCJDM67L and pCC14983A-1 harbor conjugal transfer proteins that might have roles in transmission of the T6SS; however, further studies are needed to define the mechanistic basis of T6SS mobilization in *Campylobacter* spp.

The T6SS is a versatile weapon in bacterial pathogens^15^ and exhibited contact-dependent cell cytotoxicity in *Vibrio* spp., *E. coli* and *Salmonella*^17,41^. Interestingly, the contact-dependent toxicity of the T6SS was less pronounced in a *Vibrio cholerae* clinical isolate as compared to environmental isolates^41^. The *hcp* gene product of the T6SS is thought to play a major role in cell death, and *hcp* + strains of *C. coli* and *C. jejuni* from retail chicken had increased virulence^21^. Since the T6SS is similar in chromosomal and plasmid DNA of *Campylobacter*, we speculate that the plasmid-borne T6SS functions in the virulence of *Campylobacter*. In support of this hypothesis, we show that transconjugants TCF8 and TCF11 (containing the T6SS on pCJDM202) exhibit greater hemolysis towards RBCs than the recipient *C. jejuni* NCTC11168 Nal^+^, which is a clinical isolate capable of RBC cytotoxicity. Thus, the enhanced RBC cytotoxicity in the transconjugant indicated the possible involvement of the plasmid-borne T6SS in hemolysis. Previous studies have shown that the T6SS is largely responsible for the contact-dependent cytoxicity towards RBCs^19^, which is supported by our results with transconjugants and RBC cytotoxicity. However, it is important to mention that the T6SS did not impact toxcity towards the HEK 293 cell line in this study. We also compared *C. jejuni* WP-202 and the *Δhcp3* mutant in RBC assays; a seven-day-old culture of WP2-202 showed significantly higher levels of RBC cytoxicity than the *Δhcp3* mutant; however, no significant differences were observed with three-day-old cultures. Our results using seven-day-old inoculum in RBC toxicity assays agrees with previous results^19^. The observed differences in cell cytotoxicity using older cultures might be due to increased expression of the T6SS genes in late stationary phase when nutrients are depleted.

A limitation of the current study is the failure to complement the *Δhcp3* mutant. One possibile explanation is the potential incompatibility of the vector pRY108 with pCJDM202. pRY108^31^ was derived from the cryptic *C. coli* plasmid pIP1455^42^ and was shown to replicate in *C. jejuni*, *C. coli* and *E. coli* hosts. Most shuttle vectors that are derived from pIP1455 share the same incompatibility group^42–46^. Efforts to complement the *Δhcp3* mutant are sill needed, and other vectors such as pCJ419^47^ are under consideration.

We show that pCJDM202 and pCJDM67L harbor the *tet*(O) gene that confers tetracycline resistance, which is often plasmid-mediated in *Campylobacter* spp*.*^48^. Transconjugants TCF8 and TCF11 exhibited elevated tetracycline resistance when they acquired megaplasmid pCJDM202; furthermore, the transconjugants exhibited a higher level of tetracycline resistance (up to 256 µg/ml) than the donor *C. jejuni* WP2-202 (MIC = 64 µg/ml). The recipient strain, *C. jejuni* NCTC11168, was shown to encode a functional CmeABC efflux pump responsible for multidrug resistance^35^. The inactivation of *cmeB* resulted in a 16- to 128-fold decrease in tetracycline resistance in *C. jejuni* strains containing *tet*(O)^34^. Since the *cmeABC* genes are present in the transconjugants, the elevated level of tetracycline resistance might be due to variable expression of the *tet(O)* gene borne on pCJDM202.

In conclusion, the megaplasmids described in this study encode tetracycline resistance and the T6SS, which might facilitate survival and virulence of the *C. jejuni* host. The enhanced hemolytic activity of the donor WP2-202 and transconjugants indicate a potential role for the T6SS in RBC cell cytotoxicity. It had been proposed that *Campylobacter* might use the T6SS to lyse blood cells and then use blood components as a nutrient source^19^. Hence, the T6SS and tetracycline resistance encoded by megaplasmids are likely factors that facilitate *Campylobacter* survival in retail meats.

## Supplementary information

Supplementary file1 (XLS 1465 kb)

Supplementary file2 (PDF 203 kb)
