# Supplementary material for: Molecular characterization of megaplasmids encoding the type VI secretion system in Campylobacter jejuni isolated from chicken livers and gizzards
Source: Sci Rep. 2020 Jul 27;10:12514. doi: 10.1038/s41598-020-69155-z (PMC7385129; doi:10.1038/s41598-020-69155-z)

Supplementary figure

Molecular characterization of megaplasmids encoding the Type VI secretion system in *Campylobacter jejuni* isolated from chicken livers and gizzards

Daya Marasini[1], Anand B. Karki[1], John M. Bryant[1], Robert J. Sheaff[2], and Mohamed K. Fakhr[1#]

1Department of Biological Science, The University of Tulsa, Tulsa, Oklahoma, United States of America.

2Department of Chemistry and Biochemistry, The University of Tulsa, Tulsa, Oklahoma, United States of America.

# mohamed-fakhr@utulsa.edu

Figure S1. Bar diagram showing the viability of HEK 293 cells after incubation for 6 hrs with the trans-conjugants. 11168 = NCTC11168 Nal+, TCF8 and TCF11 = trans-conjugants.

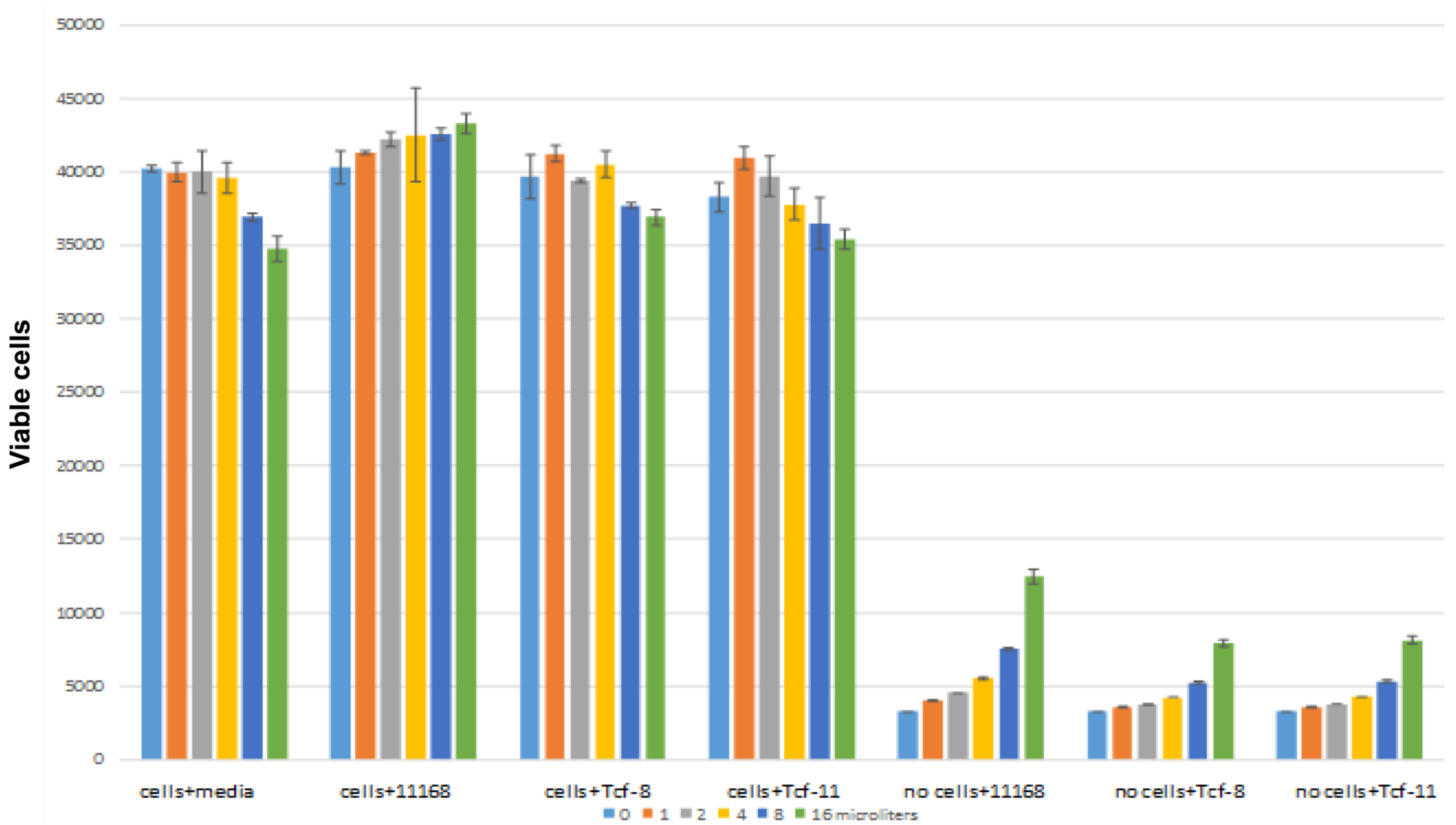

Supplement: Supplementary file 2 — Supplementary file2 (PDF 203 kb) [file 41598_2020_69155_MOESM2_ESM.pdf]
